# Potential of earlier primary care health checks for prevention of cardiovascular events in younger age groups: population-based study in the United Kingdom

**DOI:** 10.1186/s12916-026-04657-7

**Published:** 2026-01-24

**Authors:** Catherine A. Scott, Linxin Li, Peter M. Rothwell

**Affiliations:** Wolfson Centre for Prevention of Stroke and Dementia, Nuffield Department of Clinical Neuroscience, University of Oxford, Oxford, UK

**Keywords:** Heart disease risk factors, Risk assessment, Risk factors, Algorithms, Prospective studies, Cardiovascular diseases, Epidemiology

## Abstract

**Background:**

Incidence of stroke and certain cancers is increasing at younger ages in many high-income countries, prompting healthcare systems to consider how best to improve prevention, such as earlier primary care health checks. We assessed potential barriers to the success of the current proposal in England to reduce the starting age of the 5-yearly NHS health check from 40 to 30 years.

**Methods:**

In a prospective population-based study (Oxford Vascular Study; 1/4/2002–31/3/23) of 94 567 people in a subpopulation of Oxfordshire, UK, we assessed all participants with incident acute vascular events occurring at age 30–44 years and determined the proportion of those who would have qualified for active risk management were they to have had the proposed new health check prior to their event (i.e. premorbid QRISK3-10-year absolute CV-risk ≥ 10%). We also assessed CV-risk relative to age-specific ‘ideal’ risk (QRISK3-Relative Risk (RR) score, predicted “healthy-heart-age”) and number of risk factors above recommended target.

**Results:**

During 433,797 person-years of ascertainment, 217 individuals aged 30–44 years had an incident vascular event (crude incidence rate 50/100 000 person years). Of these, 155 would have been eligible for an earlier health check. The median 10-year predicted CV risk in this group was only 2.5% (IQR = 1.1–4.8%), with 148 (95%) falling below the 10% threshold for active risk management. The median 10-year risk among the 49 women was 1.1% (IQR 0.5–2.2%), with none having a predicted risk above the 10% threshold. Yet, the mean predicted “healthy heart age” gap was 9 years(SD = 7), and 137(88%) had at least one treatable risk factor above target level.

**Conclusions:**

The majority of vascular events at age 30–44 years occur in individuals with treatable risk factors above target level, yet the vast majority had falsely reassuring premorbid 10-year CV risks that were well below the 10% threshold for treatment, potentially undermining the effectiveness of earlier primary care health checks.

**Supplementary Information:**

The online version contains supplementary material available at 10.1186/s12916-026-04657-7.

## Background

Cardiovascular disease (CVD) remains a leading cause of premature death and disability worldwide [1] and is potentially preventable with existing interventions. Despite improvements in overall CVD incidence in high-income countries, recent trends indicate a concerning increase in the incidence of stroke as well as some cancers with shared risk factors at younger ages [2–4].

One proposed response to the rising disease rates at younger ages is to introduce early routine primary care health checks, as has been proposed in England, with eligibility for the NHS Health Check (NHS-HC) to be reduced from age 40 to 30 [5]. Although the evidence base for health checks has long been debated even for older age groups [6, 7], and health checks have not been adopted in some countries [8], periodic routine health checks in primary care are provided by some health care systems as a method of identifying important risk factors for cardiovascular events [9, 10]. The NHS-HC in England is currently offered every five years to people aged 40–74 without known pre-existing CVD-related conditions (Table 1) [11]. NHS-HC participants receive an estimate of their absolute risk of having a heart attack or stroke in the next 10 years (via the QRISK3 algorithm), and are then given personalised advice and/or risk factor management depending on their 10-year QRISK3 CV absolute risk score. Those with a 10-year risk score of < 10% are categorised as “low risk”, while those with a risk score ≥ 10% are eligible for active risk management including statin treatment [12].

**Table 1:** Pre-existing conditions or risk factors which exclude patients from QRISK3 scoring and or the NHS health check program

**Conditions which exclude patient from undertaking NHS health check and QRISK3 CVD assessment:** Cerebrovascular accident—haemorrhagic or ischaemic Transient ischaemic attack Coronary heart disease—myocardial infarction or angina
**Conditions which exclude patient from NHS health check program but do not exclude from use of QRISK3 CVD assessment:** Atrial fibrillation Heart failure Peripheral arterial disease Chronic kidney disease (stage 3–5) Diabetes mellitus (Type I or II) Hypertension Familial hypercholesterolemia Prescribed statins to lower cholesterol in last 6 months Serious mental illness (psychosis, schizophrenia or bipolar disease) On learning disability register Receiving prison health checks On palliative care register Previously found to have a 20% or higher risk of developing CVD over the next 10 years

The concept of earlier health checks is not an unreasonable response to rising disease rates among younger age groups, but the evidence base for health checks in younger age groups is currently limited [13], (Additional file: Table S1) [13–17]. Moreover, there are few contemporary data on the extent to which current treatment thresholds based on 10-year absolute CV risk might be a barrier to the effectiveness of prevention of cardiovascular events in younger age groups [18], or on any potential for unintended harm from false reassurance by low predicted absolute CV risks. Ideally, a high-quality randomised trial would be conducted to assess if the benefits of early health checks outweigh harm, but as cardiovascular events in the young are rare, such a trial would have to be very large and lengthy or rely on surrogate outcome markers. In the absence of such a trial, we sought to assess if early health checks have the potential to substantially reduce early-onset cardiovascular events if current risk-based treatment thresholds are applied. We assessed all individuals who had a first cardiovascular event at age 30–44 years in a prospective population-based incidence study (Oxford Vascular Study) of all patients registered in eight primary care practices in Oxford, UK. Using the proposed earlier NHS-HC as an example, we determined what proportion of those participants who would have been eligible for the proposed new health check prior to their event would have fallen above the current treatment threshold for preventative therapy based on 10-year predicted CV risk versus other representations of CV risk prediction.

## Methods

### Study population and case ascertainment

The design of the Oxford Vascular Study (OXVASC) has been previously described [19]. Briefly, OXVASC is a long-term prospective population-based study of all acute vascular events in a population of 94,567 individuals registered with nine primary care practices (roughly 100 primary care doctors) in Oxfordshire, UK. Based on ONS Census data, in 2011 (the mid-point of this study), 16.4% of the total resident population of Oxfordshire and 36.4% of Oxford city’s population were from an ethnic minority (non “white-British”) background, compared with 19.5% across England and Wales [20].

Multiple sources were used to achieve near-complete prospective identification of patients presenting to medical attention with any possible vascular event: (1) all participating primary care doctors were requested to refer patients who presented with new sudden onset transient neurological symptoms to a daily study specific TIA and stroke clinic, which provided urgent clinical investigation and treatment; (2) research clinicians also made daily visits to the emergency department of the single referral hospital in the region; (3) daily visits to the hospital’s acute stroke unit, neurology wards, and coronary care unit; (4) daily visits to bereavement officers; (5) regular searches of hospital computerised diagnostic records for patients with symptoms of vascular events; (6) regular searches of records of requests for brain or neurovascular imaging; (7) daily searches of lists of all patients in whom a troponin-I level had been requested; (8) monthly searches of primary care computer records for vascular diagnoses. Written informed consent was obtained from all patients, or assent was obtained from relatives of patients with dementia or dysphasia. Patients who had an event while temporarily away from Oxfordshire were included on their return, but visitors to Oxfordshire not normally registered with a study general practice were excluded. If a patient died before assessment, an eyewitness account of the clinical event and any relevant clinical records were reviewed.

All patients with suspected vascular events were assessed as soon as possible after seeking medical attention by a study clinical fellow in hospital, the study clinic or at home. This face-to-face interview and clinical assessment incorporated the collection of all the data which are included in an NHS-HC: demographic data, past medical history, premorbid use of preventive medication, premorbid risk factors, and family history, which were cross-referenced with lifelong medical records from primary and secondary care from which all premorbid blood pressure and cholesterol measurements were also extracted. All diagnoses were reviewed by senior study clinicians.

Acute vascular events were sub-classified by territory as described previously into cerebrovascular events (stroke and TIA), cardiac events (myocardial infarction and sudden cardiac death) and acute peripheral arterial events (PAD) (affecting the aorta, a limb, or an organ other than the heart, brain, or eye) [19]. Non-fatal and fatal acute coronary events were defined using published criteria based on the availability of history, ECG findings, cardiac biomarkers, autopsies, or death certificates [21]. Sudden cardiac deaths were coded according to recommendations for epidemiological studies [21], and needed a definite history of preceding symptoms consistent with acute coronary ischaemia, or post-mortem evidence of either clinically significant coronary atherosclerosis or acute thrombosis, or a recorded myocardial infarction during the previous 28 days.

### Analysis

For the current analyses, first-ever incident acute vascular events in participants aged 30 to 44 ascertained between 01/04/2002 and 31/03/2023 were included. This age group was specifically chosen to include individuals who would have been invited to the proposed younger health check in the 5 years prior to their event (health checks in England are currently offered to individuals from age 40 to 74 every five years).CV risk assessment (QRISK3 CV risk score) calculation was based on risk factor data collected by study physicians at the time of the acute event. Methods for QRISK3 variable calculations were based on those published by QRISK3 and are detailed in full in Additional file: Table S2 [22]. Seventeen of the 21 QRISK3 risk variables were collected during study-specific face-to-face interviews and clinical exams soon after the vascular event; history of erectile dysfunction, premorbid blood pressure readings and cholesterol measurements were collected from primary care records. Where lipids had not been measured prior to the event, data obtained at assessment were used as a proxy if prior to any lipid lowering therapy. Where total cholesterol measurement was available but HDL was not (*n*= 4 cases), the HDL was imputed based on the median value of the other participants (1.2 mmol/L), and the impact of this was explored in sensitivity analysis. Only blood pressure values prior to any vascular events were used for risk scoring. Blood pressure variability (standard deviation) was calculated from all previous readings from all medical records during the 5 years prior to the event if multiple measurements had been recorded. In routine NHS-HCs in clinical practice, some of the QRISK3 variables are often missing [23], therefore we did not impute values for missing variables, as data may not be missing at random, and imputation might introduce bias. However, we calculated and compared the estimated QRISK3 scores for the excluded cases, for included cases with missing variables, and with those with complete data.

People diagnosed with certain pre-existing clinical conditions are not eligible for an NHS-HC as they already undergo routine cardiovascular risk factor monitoring (Table 1). Eligibility for an NHS-HC was assessed by identifying all those with such conditions. The following premorbid QRISK3 scores were calculated for each participant by C.A.S. using the QRISK3 algorithm, version 2018.0, using the risk calculator at https://qrisk.org: a. QRISK3 10-year CV risk score (%). b. QRISK relative risk (QRISK3 score divided by the score of a “healthy person” defined as someone of the same age, sex and ethnic group, with no adverse clinical indicators and a total cholesterol/HDL ratio of 4.0, a stable systolic blood pressure of 125 mmHg, and BMI of 25) [22, 24, 25], c. QRISK3 Healthy Heart Age (the age at which a “healthy person” of the same sex and ethnicity has that individual’s 10-year QRISK3 score). The heart age gap was calculated by subtracting the participants’ chronological age at the time of their event from their calculated Healthy Heart Age. QRISK3-lifetime cardiovascular risk (a person's risk of developing a heart attack or stroke in their remaining lifetime to age 99) was also calculated for each participant using version 3 of the QRISK3-lifetime risk calculator (https://qrisk.org/lifetime/) [26]. Predicted risk scores in those who would have been eligible for an NHS-HC prior to their event were explored by the following predefined variables: age category (30–39 vs 40–44), sex (self-reported), ethnicity (self-reported at face-to-face interview), type of vascular event, study period (2002–2012 vs 2012–2023), deprivation (Index of Multiple Deprivation categorised as ≥ 50th centile vs < 50th centile for England), completeness of data (missing BP variability/missing any other variables/complete data) and stratified by the number of risk factors for non-attendance at NHS-HC (male sex, deprivation, smoking) [5]. Secondary analyses using the same methods were also performed in those who would not have been eligible for the NHS-HC.

This study is reported in accordance with the Strengthening the Reporting of Observational Studies in Epidemiology (STROBE) guidelines (Additional file 3).

### Patient and public involvement

This study was informed by the primary research priority identified by stroke survivors in partnership with the James Lind Alliance and the Stroke Association; “what are the best interventions to stop stroke occurring for the first time?” [27]*.* We discussed results and sought advice on the interpretation of our findings from members of the Wolfson Centre for the Prevention of Stroke and Dementia (CPSD) research PPIE group (which includes survivors of stroke and cardiovascular disease, carers and charity sector representatives) at meetings on 4.10.2024 and 13.1.2025.

### Role of the funding source

The funders had no role in the study design, collection, analysis, or interpretation of data or decision to publish the report.

## Results

**Fig. 1:**
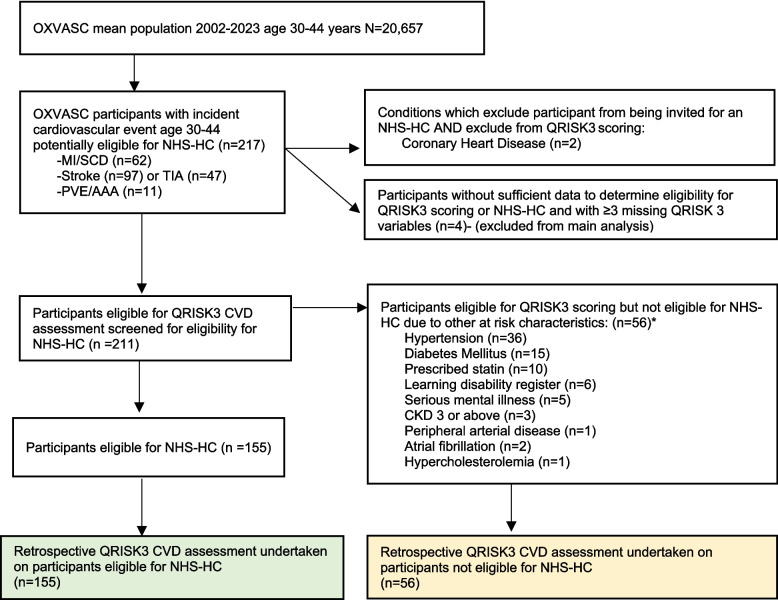
Flow diagram of participants. *Some participants had more than 1 reason they were not eligible. NHS-HC-new National Health Service Health Check, MI myocardial infarction, SCD sudden cardiac death, TIA transient ischaemic attack, PVE peripheral vascular event, AAA abdominal aortic aneurism, CKD chronic kidney disease

During 433 797 person-years of ascertainment, 217 individuals aged 30–44 years had an incident vascular event (crude incidence rate 50/100 000 person-years; Fig. 1). Two patients (0.9%) were not eligible for either QRISK scoring or an NHS-HC as they had pre-existing angina. Eligibility for the NHS-HC was uncertain in a further four (2%) who could not be assessed face-to-face and had eligibility variables missing in both primary and secondary care records.

Of the remaining 211 patients, 142 had an incident cerebrovascular event, 58 had a cardiac event (myocardial infarction or sudden ischaemic cardiac death), and 11 had an acute vascular event in another territory (Table 2). Fifty-six patients (27%) were already on one or more existing NHS screening programmes or on statin therapy prior to their event, making them ineligible for the NHS-HC (Fig. 1/Table 1). Results of risk scores for those who were not eligible for the health check are available in additional file Table S3.

**Table 2:** Baseline characteristics of study participants

Characteristics	Eligible for proposed new NHS health check	Not eligible for the proposed new NHS health check	Total^b^
No of patients	155	56	211
Age (years), mean (SD)	38.8 (4.1)	39.6 (4.3)	39.0 (4.2)
Sex			
Male	106 (68)	31 (57)	137 (65)
Female	49 (32)	25 (43)	74 (35)
Type of incident cardiovascular event			
Stroke	67 (43)	27 (48)	94 (45)
TIA	37 (24)	11 (20)	48 (23)
Cardiac (MI or sudden cardiac death)	45 (29)	13 (23)	58 (27)
Acute peripheral vascular event	6 (4)	5 (9)	11 (5)
Ethnicity			
White	127 (82)	50 (89)	177 (84)
Other	30 (19)	6 (11)	36 (17)
Time period			
2002–2012	80 (52)	25 (45)	106 (50)
2012–2023	75 (48)	31 (55)	105 (50)
Deprivation (mean IMD rank)	22,518	23,147	22,682
Non demographic QRISK3 risk factors:			
Cholesterol ratio (TC/HDL, mean (SD))	4.6 (1.5)	4.8 (1.3)	4.7 (1.4)
Systolic BP (mean (SD))	128 mmHg (15.9)	137 mmHg (18.5)	131 mmHg (17.1)
Systolic BP variability (mean (SD))	10.25 (6.08)	14.58 (5.84)	11.49 (6.31)
BMI (mean)	27 kg/m^2^ (5.6)	31 kg/m^2^ (6.7)	28 kg/m^2^ (6.1)
Diabetes (type I or II)	0	16 (29)	16 (8)
FH of angina/MI < 60 years	33 (21)	7 (13)	40 (19)
CKD 3 +	0	3 (5)	3 (1.4)
Atrial fibrillation	0	0	0
Treated hypertension	0	26 (46)	26 (12)
Migraine	48 (31)	17 (30)	65 (31)
Rheumatoid	1 (0.6)	0	1 (0.5)
Systemic lupus erythematosus	0	1 (1.8)	1 (0.5)
Severe mental illness	6 (4)	6 (11)	12 (6)
Atypical antipsychotic	0	5 (9)	5 (2)
Regular steroid medication	4 (3)	0	4 (2)
Erectile dysfunction	1 (0.6)	1 (1.8)	2 (0.9)
Uncontrolled treatable risk factors:			
Total cholesterol ≥ 5.0 mmol/l or 190 mg/dl	87 (64)	35 (67)	122 (65)
Total cholesterol ≥ 7.5 mmol/l or 290 mg/dl	5 (3)	3 (5)	8 (4)
Latest premorbid systolic BP ≥ 140 mmHg	38 (28)	23 (44)	61 (32)
BMI ≥ 25.0	97 (64)	45 (83)	142 (69)
Current smoker^a^	73 (47)	19 (35)	92 (44)
At least one of above uncontrolled treatable risk factor^c^	137 (88)	52 (93)	189 (90)

Data are numbers (%) unless specified otherwise

*SD* standard deviation

^a^Includes light/moderate/heavy smokers and those who stopped smoking less than 4 weeks prior to the vascular event

^b^Smoking data was missing for 2 participants, 23 participants did not have a premorbid systolic blood pressure reading, BMI was not available for 5 participants, and total cholesterol was not available for 28 participants

^c^One hundred sixty-two out of 211 participants had complete data for all four of these risk factors

Of the 155 patients who would have been eligible to be invited to the proposed new NHS-HC, 37 (24%) had at least one of the 21 QRISK3 score variables missing, and a further 30 (19%) had insufficient prior documented measurements of systolic blood pressure to calculate variability. The median predicted 10-year QRISK3 scores were similar for those with complete and incomplete data (additional file Table S4). Most (137/155(88%)) participants had at least one uncontrolled treatable risk factor, with 5 (3%) fulfilling criteria for screening for familial hypercholesterolemia (Table 2). The overall median 10-year QRISK3 score in the 155 eligible participants was 2.5% (IQR 1.1–4.8%), and was highest in men, those from more deprived areas, and in white patients (Table 3). The predicted risk also increased with the number of risk factors for non-attendance at NHS-HC (*p* < 0.001; Table 3).

**Table 3:** Premorbid risk scoring results by type of vascular event, age, time period, sex, and demographic characteristics. QRISK3 CVD Score; the 10-year risk score, Relative risk score: the score of a “healthy” age, sex and ethnic-matched individual, i.e. with no adverse clinical indicators and a cholesterol ratio of 4.0, a stable systolic blood pressure of 125, and a BMI of 25. “Healthy Heart Age” the age at which a “healthy person” (as above) would have the same QRISK CVD Score, and QRISK Lifetime risk score; estimated risk of getting cardiovascular disease by age 99

	***n***	**Median (IQR) predicted 10 year CVD QRISK3 score**	**QRISK < 10**	**QRISK ≥ 10**	**Mean (SD) Relative risk (RR)**	**RR ≤ 1**	**RR > 1**	**Mean (SD) additional years predicted by “health heart age”**	**Mean (SD) Lifetime risk score (to age 99)**	***n* (%) with lifetime risk > 50%**^a^
**Eligible for NHS-HC:**	**155**	**2.5% (1.1–4.8%)**	**148 (95%)**	**7 (5%)**	**3.0 (2.5)**	**17 (11%)**	**138 (89%)**	**8.9 (7.2)**	**52.3% (13.6)**	**76 (49%)**
Age 30–39	79	1.1% (0.6–3.0%)	76 (96%)	3 (4%)	3.2 (3.1)	14 (18%)	65 (82%)	7.9 (7.8)	50.8% (13.8)	35 (44%)
Age 40–44	76	3.9% (2.4–5.7%)	72 (95%)	4 (5%)	2.8 (1.8)	3 (4%)	73 (96%)	9.9 (6.4)	53.9% (13.2)	41 (54%)
Male	106	3.6% (1.6–5.3%)	99 (93%)	7 (7%)	3.3 (2.7)	7 (7%)	99 (93%)	9.9 (7.0)	55.3% (13.0)	60 (57%)
Female	49	1.1% (0.5–2.2%)	49 (100%)	0%	2.3 (2.0)	10 (20%)	39 (80%)	6.6 (7.0)	45.8% (12.6)	16 (33%)
Cerebrovascular	104	1.8% (0.9–4.2%)	101 (97%)	3 (3%)	2.9 (2.6)	12 (12%)	92 (88%)	8.1 (7.3)	50.8% (13.7)	47 (45%)
Cardiac/peripheral	51	4.3% (1.9–6.3%)	47 (92%)	4 (8%)	3.3 (2.4)	5 (10%)	46 (90%)	10.3 (6.8)	55.4% (13.0)	29 (57%)
2002–2012	75	3.0% (1.3–5.0%)	70 (93%)	5 (7%)	3.1 (2.3)	9 (12%)	66 (88%	9.6 (7.0)	54.2% (13.2)	42 (58%)
2012–2023	80	2.0% (0.9–4.4%)	78 (97%)	2 (3%)	2.9 (2.7)	8 (10%)	72 (90%)	8.2 (7.4)	50.4% (13.5)	33 (41%)
Most deprived^b^	23	3.4% (1.2–7.0%)	20 (87%)	3 (13%)	3.4 (2.3)	1 (4%)	22 (96%)	11.2 (7.4)	52.7% (10.2)	12 (52%)
Less deprived	132	2.3% (1.1–4.6%)	128 (97%)	4 (3%)	2.9 (2.6)	16 (12%)	116 (88%)	8.5 (7.1)	52.3% (14.1)	64 (48%)
White	127	2.7% (1.1–4.8%)	121 (95%)	6 (5%)	3.1 (2.5)	14 (11%)	113 (89%)	9.3 (7.4)	52.4% (13.4)	62 (49%)
Other ethnicity	28	1.6% (0.6–4.5%)	27 (96%)	1 (4%)	2.5 (2.4)	3 (11%)	25 (89%)	7.0 (5.7)	52.1% (14.4)	13 (50%)
No of risk factors for non-attendance^c^										
0	29	1.1% (0.4–1.9%)^#^	100%	0%	1.5 (0.8)	34%	66%	3.5 (4.8)	43.1% (12.2)	7 (24%)
1	62	1.9% (0.9–3.8%)	100%	0%	2.4 (1.9)	10%	90%	7.1 (5.5)	51.5% (11.4)	30 (48%)
2	53	4.4% (2.1–6.2%)	49 (92%)	4 (8%)	4.3 (3.3)	1 (2%)	52 (98%)	12.7 (7.5)	57.9% (14.2)	33 (62%)
3	11	6.8% (4.2–10.3%)	8 (73%)	3 (27%)	4.2 (2.0)	0%	100%	14.8 (5.8)	54.4% (12.5)	6 (55%)
**Not eligible for NHS-HC**	**56**	**5.5% (2.4–10.2%)**	**42 (75%)**	**14 (25%)**	**7.3 (7.0)**	**3 (5%)**	**53 (95%)**	**17.4 (10.4)**	**57.0% (12.5)**	**39 (70%)**
**All**	**211**	**3.2% (1.2–5.8%)**	**190 (90%)**	**21 (10%)**	**4.2 (4.6)**	**20 (9%)**	**191 (91%)**	**11.2 (9.0)**	**53.6% (13.4)**	**115 (55%)**

^#^Non parametric test for trend *p* < 0.001

^a^The authors of QRISK lifetime have previously published that a 50% lifetime risk is at the 90th centile in the derivation population

^b^IMD most deprived 50th centile England

^c^Risk factors for non-attendance: male, deprivation, and smoker

**Fig. 2:**
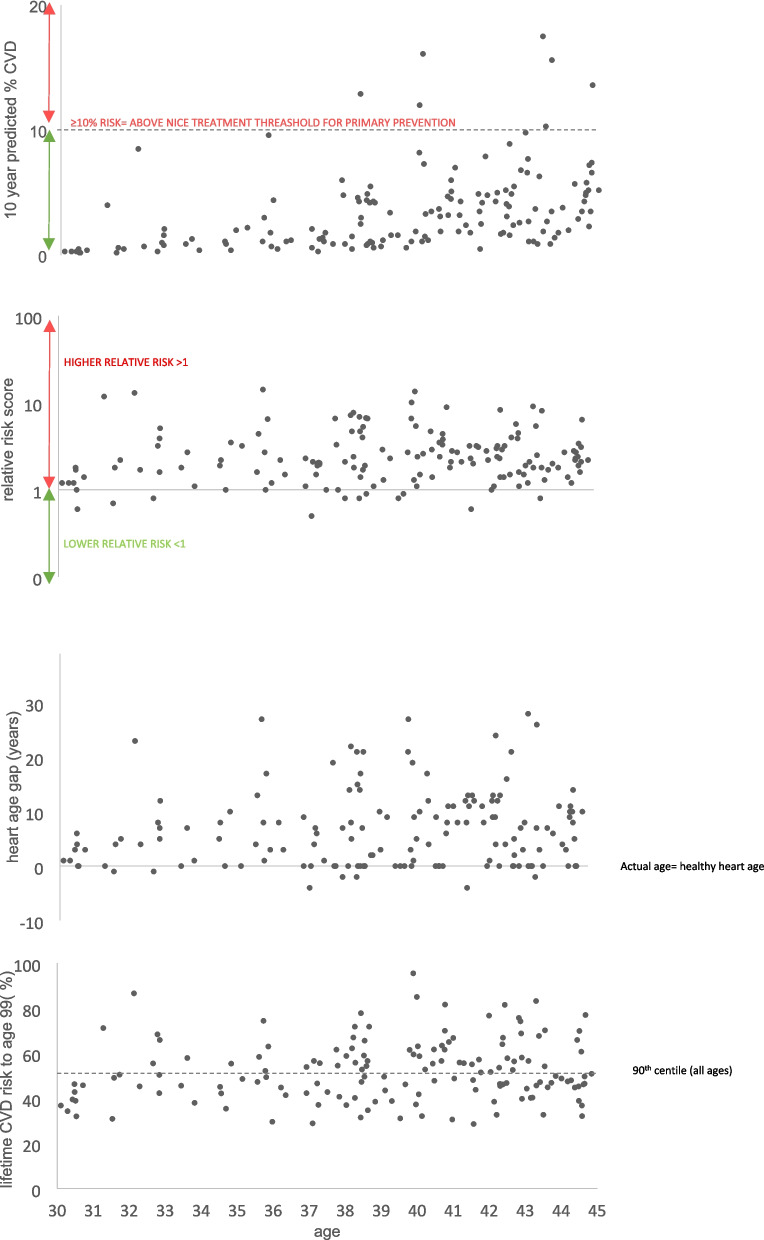
Premorbid 10 year predicted CVD QRISK3 score compared with alternative QRISK predicted risk scores in all patients who had a vascular event aged 30–44 years in those who would be eligible for an NHS health check by age (*n* = 155)

The 10-year cardiovascular risk categorisation of the 155 eligible patients was ‘low’ (< 10%) in 148 (95%); 7(5%) had a risk score between 10 and 20%, and none had a predicted 10-year risk > 20% (Table 3, Fig. 2). All eligible females were classified as low risk, and only 7% of males had risk scores above the 10% threshold. The 4 additional excluded patients in whom eligibility for the NHS-HC was uncertain had estimated QRISK3 scores < 3%.

**Fig. 3:**
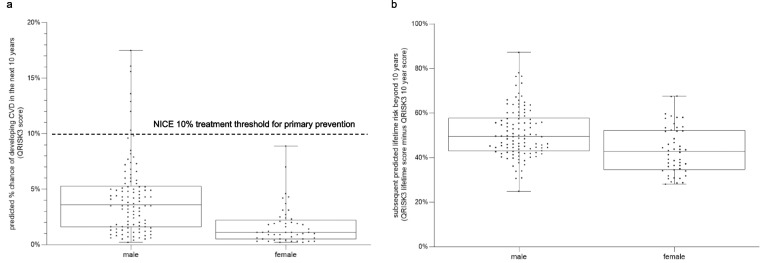
**a** Distribution of predicted 10-year CVD QRISK3 scores in 30–44 year olds who would be eligible for the new NHS health check by sex. **b** subsequent lifetime risk (QRISK Lifetime score to age 99 minus 10-year QRISK3 score) (*n* = 155). The dots represent actual data points of individual predicted risk scores; the box represents the first and third quartiles, with the central horizontal line indicating the median. The whiskers represent the lowest and highest data point

If the threshold for predicted “low risk” was reduced to 5% or 2.5%, 121/155(78%) and 77/155(50%) of participants respectively would still fall under these lower thresholds. However, using the predicted Relative QRISK3 score (RR) > 1 identified 138 (89%) patients to be at greater than their age, sex and ethnicity-adjusted ‘ideal’ risk, including 99 (93%) males and 39 (80%) females (Fig. 3 and S1/Table 3). Similarly, the predicted “Healthy Heart Age” was greater than the participants’ actual age in 140 (90%) patients, with 62 (40%) having a heart age gap of at least 10 years, and 76 (49%) having a predicted QRISK3 lifetime risk of > 50% (the reported 90th centile in the derivation dataset [21]) (Fig. 2).

In terms of the disparity in risk scores between male and female patients, the median absolute predicted cardiovascular 10-year risk for males was 3 times higher than that of females (Fig. 3a), but this was reduced to only 1.2 times higher when comparing the subsequent lifetime risk thereafter (i.e. lifetime risk minus 10-year risk; Fig. 3b). The sex difference was also reduced when risk was expressed in terms of the Relative Risk or Healthy Heart Age (Table 3). Two illustrative cases of female patients are summarised in Additional file 2.

## Discussion

The vast majority of patients who had acute cardiovascular events in our study and who would have been eligible for the proposed new younger NHS-HC at 30–39 years, including all women, had pre-morbid 10-year cardiovascular risks well below the 10% treatment threshold for active risk management, although most had risks that were also well above the ‘ideal’ level. Even among those who were already known to have pre-existing CVD-related conditions (i.e. already on a screening register), the majority (75%) were still below the 10% treatment threshold.

Few studies have reported premorbid vascular risk scores in younger adults with acute cardiovascular events. A retrospective study of patients with myocardial infarction (MI) at age < 50 years in two US hospitals from 2000 to 2016 reported a median predicted 10-year risk of 4.8% using the Atherosclerotic Cardiovascular Disease (ASCVD) Risk Score [28]. A similar US study reported a median 10-year pre-morbid ASCVD risk of 6.4% in patients aged 40–54 with a first MI between 1995 and 2012 [29]. Our findings in all patients aged 30–45 years with acute cardiac events were similar (median 10-year CV risk = 5.0%).

The predicted 10-year CV risk in our study was particularly low in women (median = 1.1%, IQR = 0.5–2.1%). In the US study of patients with MI at age < 50 years, there was also a trend towards lower predicted risk in women versus men [28]. Clearly, the extent of the sex difference in vascular risk in younger populations will depend on the time horizon of the prediction, as was evident in our analyses of longer-term risk.

Some would argue that early health checks are required in order to reap the expected long-term benefits of early control of major vascular risk factors, thereby reducing the problem of residual risk following treatment initiated at older ages [30]. However, our data highlight two barriers to improving prevention of CV events at younger ages based on the current predicted risk-based paradigm—the relative lack of predictive power of current models in young adults—and the fact that only a tiny proportion of young adults with vascular risk factors exceed the current 10% 10-year CV-risk threshold for treatment.

The relative lack of predictive power of current models in young adults means that the 10-year absolute risk of vascular events is inevitably low even in patients who appear likely to have a high lifetime risk (Additional file 2). Trials of treatment of a single risk factor, such as moderate hypertension or hyperlipidaemia, in otherwise unselected younger patients consequently yield large numbers needed to treat (NNT) to prevent events over the next few years [31, 32]. Even trials of strict control of multiple cardiovascular risk factors at younger ages will likely require long-term treatment with multiple medications to yield a clinically impactful reduction in absolute CV risk [33]. Another barrier to risk prediction and risk reduction using current strategies at younger ages is the significant residual risk due to the high proportion of non-atherosclerotic aetiologies, particularly in younger women in whom stroke accounted for the majority of CV events.

Future improvements in risk prediction at younger ages may be possible. The forthcoming update of QRISK3 (QR4) will add seven new risk variables, including female-specific factors [34], but only 12 (8%) of the 155 individuals eligible for the new health check in our study had any of these new variables (Additional file 1: Table S5). Incorporation of other risk factors that are more specific to risk at younger ages will also be required, such as family history of premature stroke rather than only premature coronary events [35]. Developing models using AI may improve prediction by incorporating intensity, timing and duration of exposure to risk factors and interventions, and including non-linear and time-dependent exposure-risk associations as well as complex interactions with age and sex, provided appropriate detailed training data is used. Future algorithms may also need to incorporate biomarkers such as Lp(a) as well as imaging markers and genomic sequences, but both their development and their application in practice will require improvements in detail and linkage of electronic medical records. As algorithms become more complex, their automated incorporation into electronic patient records will be vital.

The second issue highlighted by our data, the need to adapt risk thresholds at younger ages, is now already increasingly recognised, with three possible approaches advocated. First, to reduce the risk thresholds of short-term risk prediction in younger individuals, the European Society for Cardiology SCORE2 now defines low risk as < 2.5% 10-year risk in those under 50 years, and < 5% in those over 50 years [36]. However, this score is not currently intended for use in those aged under 40., Basing treatment thresholds on > 75th or ≥ 90th centile of age and sex-specific short-term risk offers another pragmatic route to reduce false reassurance of younger individuals but relies on normal ranges/centiles being readily available [37].

The second approach is to use risk measures relative to an optimal health state, such as the QRISK3 Healthy Heart Age or Relative Risk, which are easier to communicate to patients, but there are concerns that they could lead to overtreatment if used for treatment decisions [38]. The third approach is to expand the horizon of risk prediction at younger ages; the new Predicting Risk of cardiovascular disease EVENT (PREVENT) calculator from the American Heart Association provides both a 10 year and 30 year risk [39], NICE guidelines suggest a lifetime risk tool, such as QRISK3-lifetime, be used to motivate lifestyle changes, particularly for people with a 10‑year QRISK3 score less than 10%, and people under 40 who have CVD risk factors [25]. While our results support this idea, with half of our patients having a lifetime risk score above the 90th centile reported by the QRISK lifetime publication [26], there is no consensus regarding thresholds for high 30 year or lifetime risk. The Primary Prevention of Subclinical Atherosclerosis in Young Adults (PRECAD) trial has selected patients aged 20 to 39 years with a 30 year risk of 30% or over [33], while others suggest the 75th centile [40]. Young patients with higher measures of relative or longer term risk may be more willing to engage in intensive lifestyle intervention, earlier initiation of lipid or blood pressure lowering therapies with lower targets, or could be offered more frequent risk assessment. However, it is vital that low short-term risks are not a barrier to treatment of individual risk factors in young people, such as blood pressure or lipids, which were raised in many of the participants in our study despite the low short-term predicted vascular risk.

Our study focussed on the potential impact of short-term absolute risk prediction as a barrier to prevention of vascular events occurring in those age 30–45, but it is important to consider that 10-year CV risk does not reflect all of the long-term morbidity that is attributable to treatable cardiovascular risk factors. For example, early midlife hypertension is strongly related to the longer-term risks of dementia and heart failure [41, 42], both of which now represent as great a risk as acute manifestations of vascular disease, and often occur without a preceding acute CV event. The social and economic consequences of CV events are also often greater at younger ages. A disabling stroke in a 35-year-old might lead to the loss of 30 years of employment and taxable income for the individual, and possibly also for their partner if care is required. The state will not only lose income and other taxes, but may also potentially have to pay for long-term care.

There is also a potential unintended risk of harm from the proposed new NHS-HC for younger adults. The vast majority of patients in our study would likely be told that their premorbid risk was too low to merit treatment. This could give false reassurance and might therefore reduce the likelihood that they would adopt a healthier lifestyle. There was possible evidence of such an effect in the Inter99 trial of health checks in Denmark, with an increase in mortality in women in areas in which uptake of the checks was high [43]. Our data support the use of longer horizon absolute or relative risk measures to reduce this potential harm.

Although OXVASC is the only population-based study of all acute vascular events irrespective of age, and thus provides an ideal opportunity to assess the impact of the proposed changes to population-based cardiovascular screening, our study does have some limitations. First, a few of the 21 QRISK3 variables were missing in some of our patients. However, variables such as standard deviation of blood pressure are rarely calculated in routine practice, and the original QRISK3 derivation cohort had complete data for smoking, systolic blood pressure, BMI, and cholesterol ratio in only 27% of the sample [24]. Second, our CV risk assessment calculation was done retrospectively, but we used risk factor data, such as blood pressures, that were recorded prior to the event, and only lipid levels at the time of the event were used if prior measures were not available. Moreover, prospective ascertainment of almost all patients in the study population with an event, including out-of-hospital deaths, minimised selection biases. Third, as we did not perform the health check on the underlying population, we were not able to assess the utility of the NHS-HC in those young people who did not have vascular events. Fourth, although the OXVASC population includes a wide range of deprivation and has a rural/urban mix and ethnicity that mirrors the UK, CV risk will be higher in some other UK regions. On the other hand, CV risk is likely to be similar or lower in many other high-income countries [1]. Fifth, we present results stratified by the index of multiple deprivation (IMD) while the QRISK3 score uses the Townsend score to measure deprivation. We chose to analyse the results using the IMD score as it includes 8 variables, rather than the 4 used in the Townsend score. Finally, it is important to consider the NHS-HC also screens for diabetes, CKD, familial hyperlipidaemia, alcohol excess, and may still help some people adopt healthier behaviour, including smoking cessation, which could impact longer-term cardiovascular risk. We focussed only on the cardiovascular risk assessment component of the NHS-HC as a potential solution to the rising stroke risk in young people. However, while evidence suggests the experiences of patients who attend health checks is positive [44], making them politically popular, in considering the overall impact it should be borne in mind that fewer than half of the population takes up the offer of health checks, with affluent female non-smokers being particularly over-represented [5]. Indeed, our results confirm that those people with the highest predicted pre-morbid CV risk had the greatest number of risk factors for non-attendance.

## Conclusions

In considering how best to combat the increasing incidence of cerebrovascular events in younger adults, the seemingly reasonable approach of introducing routine health checks at younger ages is unlikely to be effective without improved risk prediction. In the meantime, the 10% 10-year CV risk threshold for active management of risk factors may be a barrier to the effectiveness of health checks in younger adults.

## Supplementary Information

Additional file 1: Table S1. Previous Studies of Screening for CVD which include and report outcomes for individuals at younger ages. Table S2. QRISK3 variables definitions and methods to determine them in OXVASC patients. Table S3. Premorbid QRISK3 risk scoring results in those who would not be eligible for the new NHS health check by type of vascular event, age, time period, sex, and demographic characteristics. Table S4. Premorbid QRISK3 risk scoring results in those eligible for the NHS health check categorised by completeness of data variables. Table S5. Prevalence of new variables included in the QR4 CVD risk score and their potential impact on premorbid risk scoring in all patients with vascular events and sufficient data to ascertain QR4 variables age 30–44. Figure S1 a. Premorbid predicted absolute 10 year % cardiovascular QRISK3 score vs relative risk score in all patients who had a vascular event aged 30–44 years in those who would be eligible for NHS health check. Figure S1 b. Premorbid predicted absolute 10 year % cardiovascular QRISK3 score vs relative risk score in all patients who had a vascular event aged 30–44 years in those who would not be eligible for NHS health check.

Additional file 2. Case Vignettes.

Additional file 3. STROBE Statement—checklist.

## Data Availability

Relevant data for the current analysis is provided within the manuscript and supplementary information files. Data requests will be considered by the Oxford Vascular Study (OXVASC) Study Director (PMR-peter.rothwell@ndcn.ox.ac.uk).

## Abbreviations

CVD: Cardiovascular disease
NHS-HC: National Health Service Health Check
WHO: World Health Organization
OXVASC: Oxford Vascular Study
ONS: The Office for National Statistics
TIA: Transient ischaemic attack
PAD: Peripheral arterial disease
MI: Myocardial infarction
BP: Blood pressure
CPSD: Centre for the Prevention of Stroke and Dementia
PPIE: Patient and Public Involvement and Engagement
RR: Relative risk
ASCVD: Atherosclerotic Cardiovascular Disease Risk Score
US: United States
NNT: Number needed to treat
SCORE2: Systematic Coronary Risk Evaluation 2 Score
PREVENT: Predicting Risk of Cardiovascular Disease Events Score
BMI: Body mass index
UK: United Kingdom
IMD: Index of multiple deprivation
CKD: Chronic kidney disease
