# Supplementary material for: Potential of earlier primary care health checks for prevention of cardiovascular events in younger age groups: population-based study in the United Kingdom
Source: BMC Med. 2026 Jan 24;24:107. doi: 10.1186/s12916-026-04657-7 (PMC12914909; doi:10.1186/s12916-026-04657-7)
Supplement: Supplementary file 1 — Additional file 1: Table S1. Previous Studies of Screening for CVD which include and report outcomes for individuals at younger ages. Table S2. QRISK3 variables definitions and methods to determine them in OXVASC patients. Table S3. Premorbid QRISK3 risk scoring results in those who would not be eligible for the new NHS health check by type of vascular event, age, time period, sex, and demographic characteristics. Table S4. Premorbid QRISK3 risk scoring results in those eligible for the NHS health check categorised by completeness of data variables. Table S5. Prevalence of new variables included in the QR4 CVD risk score and their potential impact on premorbid risk scoring in all patients with vascular events and sufficient data to ascertain QR4 variables age 30–44. Figure S1 a. Premorbid predicted absolute 10 year % cardiovascular QRISK3 score vs relative risk score in all patients who had a vascular event aged 30–44 years in those who would be eligible for NHS health check. Figure S1 b. Premorbid predicted absolute 10 year % cardiovascular QRISK3 score vs relative risk score in all patients who had a vascular event aged 30–44 years in those who would not be eligible for NHS health check. [file 12916_2026_4657_MOESM1_ESM.docx]

**ADDITIONAL FILE 1 –Supplementary tables and figures**

**Table S1** Previous Studies of Screening for CVD which include and report outcomes for individuals at younger ages (adapted from the WHO Health Evidence Network Synthesis Report, 2024)^1^

| Study, year, setting | Description |
| --- | --- |
| **DanMonica, 1982^2^ Copenhagen suburbs, Denmark** | |
| Design | Parallel-group RCT |
| Age of participants | 30-60 years |
| Duration | 30 years |
| Health check Intervention | Questionnaire (smoking, diet, physical activity, alcohol, family history), clinical examination (weight, height, blood pressure, pulse, ECG, lung function, ultrasound tests and other non-invasive tests), serum lipids and urine analysis. Intervention group invited for three health checks over the 11-year study period; if needed, participants were referred to GPs. Health check did not appear to involve absolute CV risk score; measures of all risk factors and individually tailored information about lifestyle and chronic diseases; referral to hospital if further investigation was warranted. |
| Absolute CV risk score | No |
| Intervention group | 4789 |
| Control group | 12994 |
| Results | Total Mortality: HR=1.03 (95% CI 0.98-1.09) |
|  | IHD morbidity and mortality: HR=0.99 (95% CI 0.92-1.07) |
|  | Stroke morbidity and mortality HR=1.14 (95% CI 1.04-1.25) |
| Age specific results | Not presented |
| **The Ebeltoft Health Promotion Project (EHPP),1991^3^**  **Nine GPs in Ebeltoft**  **Denmark** | |
| Design | Parallel-group RCT |
| Age of participants | 30-49 |
| Duration | 24 years |
| Health check Intervention | Invitees were offered one of three general health checks regimens (a- at baseline and 1 year and 5 years, b-as per a + a 45 minute GP consultation, c-a general health check only) All participants were offered a health check at 15 years. Non-invitees in Ebeltoft received a questionnaire at baseline and were offered a general health check at year 15 (and censored). The external control group, (the remaining Danish population) received routine care only. |
| Absolute CV risk score | No |
| Intervention group | 2000 |
| Control group | 1. Internal control group 1464 2. External control group 1 511 498 Danes living outside of the municipality |
| Results | Total mortality: HR=0.93(95% CI 0.75-1.16)  CVD morbidity and mortality: HR=0.93(95% CI 0.88-1.41) |
| Age specific results | Not presented |
| **Inter99, 1999 ^4^ Western Copenhagen** |  |
| **Denmark** |  |
| Design | Parallel group RCT |
| Age of participants | 30-60 years |
| Duration | 10 years |
| Health check Intervention | Invitation to screening, risk assessment and lifestyle counselling; those at high risk were offered 6 sessions of lifestyle counselling and re-invited after 1 and 3 years. All participants were re-invited for final screening, counselling and maintenance planning at 5 years |
| Absolute CV risk score | Yes- 10 year risk of fatal and non-fatal ischaemic heart disease was calculated using the PRECARD score |
| Intervention group | 11 629 |
| Control group | 47 987 |
| Results | Total mortality: HR=1.00 (95% CI 0.91-1.09)  IHD morbidity and mortality: HR=1.03 (95% CI 0.94-1.13)  Stroke morbidity and mortality: HR=0.98(95% CI 0.87-1.11) |
| Age specific results | Not presented |
| **General Health Check Study 1969^5^,**  **Stockholm, Sweden** | |
| Design | Parallel-group RCT |
| Age of participants | 18-65 years |
| Duration | 22 years |
| Health check Intervention | extensive general health check over one day (health-physician, psychiatric-psychiatrist and social-social worker) |
| Absolute CV risk score | No |
| Intervention group | 3064 |
| Control group | 29122 |
| Results | Mortality: RR=1.03(95% CI 0.94-1.14)  CVD mortality RR=1.06(95% CI 0.88-1.23) |
| Age specific results | Not presented |

1. Jørgensen T, Rotar O, Juhl CB, Linneberg A. WHO Health Evidence Network Synthesis Reports. What is the effectiveness of systematic population-level screening programmes for reducing the burden of cardiovascular diseases? Copenhagen: WHO Regional Office for Europe

© World Health Organization 2024.; 2024.

2. Skaaby T, Jørgensen T, Linneberg A. Effects of invitation to participate in health surveys on the incidence of cardiovascular disease: a randomized general population study. *Int J Epidemiol* 2017; **46**(2): 603-11.

3. Bernstorff M, Deichgræber P, Bruun NH, Dalsgaard EM, Fenger-Grøn M, Lauritzen T. A Randomised Trial Examining Cardiovascular Morbidity and All-Cause Mortality 24 years Following General Health Checks: the Ebeltoft Health Promotion Project (EHPP). *BMJ Open* 2019; **9**(10): e030400.

4. Jørgensen T, Jacobsen RK, Toft U, Aadahl M, Glümer C, Pisinger C. Effect of screening and lifestyle counselling on incidence of ischaemic heart disease in general population: Inter99 randomised trial. *BMJ : British Medical Journal* 2014; **348**: g3617.

5. Theobald H, Bygren LO, Carstensen J, Hauffman M, Engfeldt P. Effects of an assessment of needs for medical and social services on long-term mortality: a randomized controlled study. *Int J Epidemiol* 1998; **27**(2): 194-8.

**Table S2** QRISK3 variables definitions and methods to determine them in OXVASC patients

| **QRISK3 variables** | **Method of ascertainment** |
| --- | --- |
| 1. Age (years) | The age the patient was at the time of their vascular event |
| 2. Sex (male/Female) | Collected from patient at face to face interview |
| 3. Ethnic origin (White or not stated, Indian, Pakistani, Bangladeshi, Other Asian, Black Caribbean, Black African, Chinese, Other ethnic group) | Collected from patient at face to face interview |
| 4. Deprivation (as measured by the Townsend score) | patient’s postcode at the time of their vascular event |
| 5. Systolic blood pressure (mmHg) | The most recent systolic blood pressure within 5 years prior to the reference date in primary or secondary care record, whichever was most recent |
| 6. Body Mass Index (BMI)- height(cm) and weight (kg) | The baseline BMI objectively measured by the study physician at the time of the vascular event. For fatal events BMI was measured at post mortem examination. |
| 7. Total cholesterol: high density lipoprotein cholesterol ratio | The most recent premorbid measurements were used when available within 5 years of event. For participants who had missing premorbid data baseline data was used as a proxy if this was within 24 hours of the vascular event and prior to any lipid lowering therapy. Where HDL was missing but Total Cholesterol was available, HDL was estimated to be 1.2mm/L as a proxy (the median from other participants HDL readings). |
| 8. Smoking status (non-smoker, former smoker, light smoker (1-9/day), moderate smoker (10-19/day), or heavy smoker (≥20/day)) | Smoking status was collected at face to face interview and cross checked with their primary care records. Former smoker was defined as a stop date at least 1 month prior to cardiovascular event. |
| 9. Family history of coronary heart disease (angina or myocardial infarction) in mother, father, brother or sister at less than 60 years (y/n) | Detailed family history questionnaire during face to face interview. |
| 10. Diabetes (type 1, type 2, or no diabetes) | Data collected from face to face interview at baseline visit and review of primary care record. * |
| 11. Treated hypertension (y/n) | Data collected from face to face interview at baseline visit and review of primary care record Diagnosis of hypertension at any time in the patient’s records AND on antihypertensive treatment if 1 or more medication requests in primary care record within 6 months prior to the reference date. |
| 12. Atrial fibrillation (including atrial fibrillation, atrial flutter, and paroxysmal atrial fibrillation) (y/n) | Data collected from face to face interview at baseline visit and review of primary care record. * |
| 13. Rheumatoid arthritis (diagnosis of rheumatoid arthritis, Felty’s syndrome, Caplan’s syndrome, adult onset Still’s disease, or inflammatory polyarthropathy not otherwise specified) (y/n) | Data collected from face to face interview at baseline visit and review of primary care record. * |
| 14. Chronic kidney disease stage 3 or higher (y/n) | Data collected from face to face interview at baseline visit and review of primary care record for CKD stage 3 or higher.* |
| 15. Migraine (y/n) | Data collected from face to face interview at baseline visit and review of primary care record. * |
| 16. Corticosteroid use (y/n) | Defined as ≥2 prescriptions, with the most recent one within the 28 days before study entry data collected from face to face interview and review of primary care prescription record. |
| 17. Systemic Lupus Erythematosus (SLE) (y/n) | Data collected from face to face interview at baseline visit and review of primary care record. * |
| 18. Atypical antipsychotic use (y/n) | Data collected from face to face interview at baseline visit and review of primary care prescription record. |
| 19. Severe mental illness (schizophrenia, bipolar disorder and moderate/severe depression) (y/n) | Data collected from face to face interview at baseline visit and review of primary care record. * |
| 20. Erectile dysfunction (y/n) | Data collected from review of primary care record. * |
| 21. Blood pressure variability (SD of systolic blood pressure) | The SAMPLE style Standard deviation of all of the available systolic blood pressures prior to the reference date recorded in the last 5 years (where there was only one reading this was left blank) |

*if a participant was first diagnosed with this condition or risk factor at the time of their event or after their event they were coded as not having the risk factor for risk scoring

**Table S3** Premorbid QRISK3 risk scoring results in those who would not be eligible for the new NHS health check by type of vascular event, age, time period, sex, and demographic characteristics. *QRISK3 CVD Score; the 10 year risk score, Relative risk score: the score of a “healthy” age, sex and ethnic matched individual, i.e. with no adverse clinical indicators and a cholesterol ratio of 4.0, a stable systolic blood pressure of 125, and BMI of 25.”Healthy Heart Age” the age at which a “healthy person” (as above) would have the same QRISK CVD Score, and QRISK Lifetime risk score;* *estimated risk of getting cardiovascular disease by age 99.*

|  | **n** | **Median 10 year CVD score (IQR)** | **10 year risk <10%** | **10 year risk ≥10%+** | **Mean Relative risk (SD)** | **RR<=1** | **RR>1** | **Mean (SD)**  **Excess Healthy Heart age (years)** | **Mean (SD) Lifetime risk score (to age 99)** | **n (%) with lifetime risk >50%*** |
| --- | --- | --- | --- | --- | --- | --- | --- | --- | --- | --- |
|  |  |  |  |  |  |  |  |  |  |  |
| **Total** | **56** | **5.5% (2.4-10.2%)** | **42 (75%)** | **14 (25%)** | **7.3(7.0)** | **3 (5%)** | **53(95%)** | **17.4 (10.4)** | **57.0%(12.5)** | **39(70%)** |
| Age 30-39 | 18 | 2.8% (1.6-7.5%) | 15(83%) | 3(17%) | 9.1(7.8) | 1(6%) | 17(94%) | 18.4 (11.8) | 54.4% (13.8) | 11(61%) |
| Age 40-44 | 38 | 6.3% (3.4-11.1%) | 27(71%) | 11(28%) | 6.5(6.6) | 2(5%) | 36(95%) | 17.0(9.7) | 58.2% (11.9) | 28 (74%) |
| Male | 31 | 6.5% (3.9-11.1%) | 22(71%) | 9(19%) | 6.9(6.4) | 0(0%) | 31(100%) | 18.2(9.1) | 62.0(11.5) | 27(87%) |
| Female | 25 | 2.7% (1.5-9.4%) | 20(80%) | 5(20%) | 7.8(7.9) | 3(12%) | 22(88%) | 16.5(11.9) | 50.8(11.0) | 12(48%) |
|  |  |  |  |  |  |  |  |  |  |  |
| Stroke | 27 | 4.2% (1.8-7.2%) | 22(81%) | 5(19%) | 7.7(7.8) | 2(7%) | 25(93%) | 17.3(11.1) | 55.0%(12.8) | 17(63%) |
| TIA | 11 | 2.8% (1.7-8.8%) | 9(82%) | 2(18%) | 4.6(4.2) | 1(9%) | 10(91%) | 13.7(10.7) | 58.2%(14.5) | 8(73%) |
| Cardiac | 13 | 9.9% (6.9-12.4%) | 7(54%) | 6(46%) | 8.2(6.0) | 0% | 100% | 21.8(8.1) | 62.0%(10.1) | 11(85%) |
| Peripheral | 5 | 5.1% (1.4-5.1%) | 4(80%) | 1(20%) | 9.2(9.9) | 0% | 100% | 15.2(10.3) | 52.5%(11.2) | 3(60%) |
| 2002-12 | 31 | 5.8% (3.0-10.3%) | 23(74%) | 8(26%) | 7.7(7.8) | 0% | 100% | 18.0(10.2) | 61.1(11.1) | 25(81%) |
| 2012-23 | 25 | 4.7% (1.8-9.8%) | 19(76%) | 6(24%) | 7.0(6.0) | 2(8%) | 23(92%) | 17.4(10.0) | 53.8(13.0) | 15(60%) |
|  |  |  |  |  |  |  |  |  |  |  |
| Most deprived ** | 10 | 9.8% (3.4-20.8%) | 5(50%) | 5(50%) | 14(9.4) | 0% | 100% | 26.7(10.1) | 58.7%(10.4) | 7(70%) |
| Least deprived *** | 46 | 5.1% (2.4-8.4%) | 37(80%) | 9(20%) | 5.9(5.5) | 3(4%) | 43(96%) | 15.4(9.4) | 56.6% (13.0) | 32(70%) |
| White | 50 | 5.8% (2.0-10.4%) | 37(74%) | 13(27%) | 7.1(6.8) | 3(6%) | 47(94%) | 17.1(10.2) | 57.0%(12.5) | 36(72%) |
| Other ethnicity | 6 | 3.2% (2.0-15.2%) | 5(83%) | 1(17%) | 9.0(9.7) | 0% | 100% | 20.2(12.5) | 56.7%(14.2) | 3(50%) |

*the authors of QRISK lifetime have previously published that a 50% lifetime risk is at the 90^th^ centile in the derivation population. ** IMD <50^th^c England *** IMD ≥50^th^c England

**Table S4** Premorbid QRISK3 risk scoring results in those eligible for the NHS health check (n=155) categorised by completeness of data variables

|  | **n** | **Median 10 year CVD score (IQR)** | **10 year risk <10%** | **10 year risk ≥10%+** |
| --- | --- | --- | --- | --- |
|  |  |  |  |  |
| All 21 QRISK variables Complete | 88 (57%) | 2.6% (1.1-4.9%) | 93% | 7% |
| 1-2 variables missing including BP variability | 67 (43%) | 2.3% (1.0-4.8%) | 99% | 1% |
| All 21 QRISK variables complete except BP variability | 118 (76%) | 2.7% (1.1-4.9%) | 94% | 6% |
| 1-2 variables missing excluding BP variability | 37 (24%) | 2.1% (1.0-4.2%) | 100% | 0% |

**Table S5** Prevalence of new variables included in the QR4 CVD risk score^1^ and their potential impact on premorbid risk scoring in all patients with vascular events and sufficient data to ascertain QR4 variables age 30-44 (n=211)

|  | **n** | **Male** | **Female** |
| --- | --- | --- | --- |
|  |  |  |  |
| Learning disability without Down’s syndrome | 4 | 2 | 2 |
| Learning disability with Down’s syndrome | 2 | 1 | 1 |
| Chronic Obstructive Pulmonary Disease (COPD ) | 1 | 1 |  |
| Oral cancer | 0 |  |  |
| Lung cancer | 0 |  |  |
| Blood cancer | 2 | 2 |  |
| Brain cancer | 4 | 2 | 2 |
| Post-natal depression | 4 |  | 4 |
| Pre-eclampsia | 8 |  | 8 |
| **Total** | **25** | **8** | **17** |
|  |  |  |  |

^1^Hippisley-Cox J, Coupland CAC, Bafadhel M, et al. Development and validation of a new algorithm for improved cardiovascular risk prediction. *Nature Medicine* 2024.

**Figure S1**

a. Premorbid predicted absolute 10 year % cardiovascular QRISK3 score vs relative risk score in all patients who had a vascular event aged 30-44 years in those who would be eligible for NHS health check (n=155)

NICE 10% treatment threshold for primary prevention


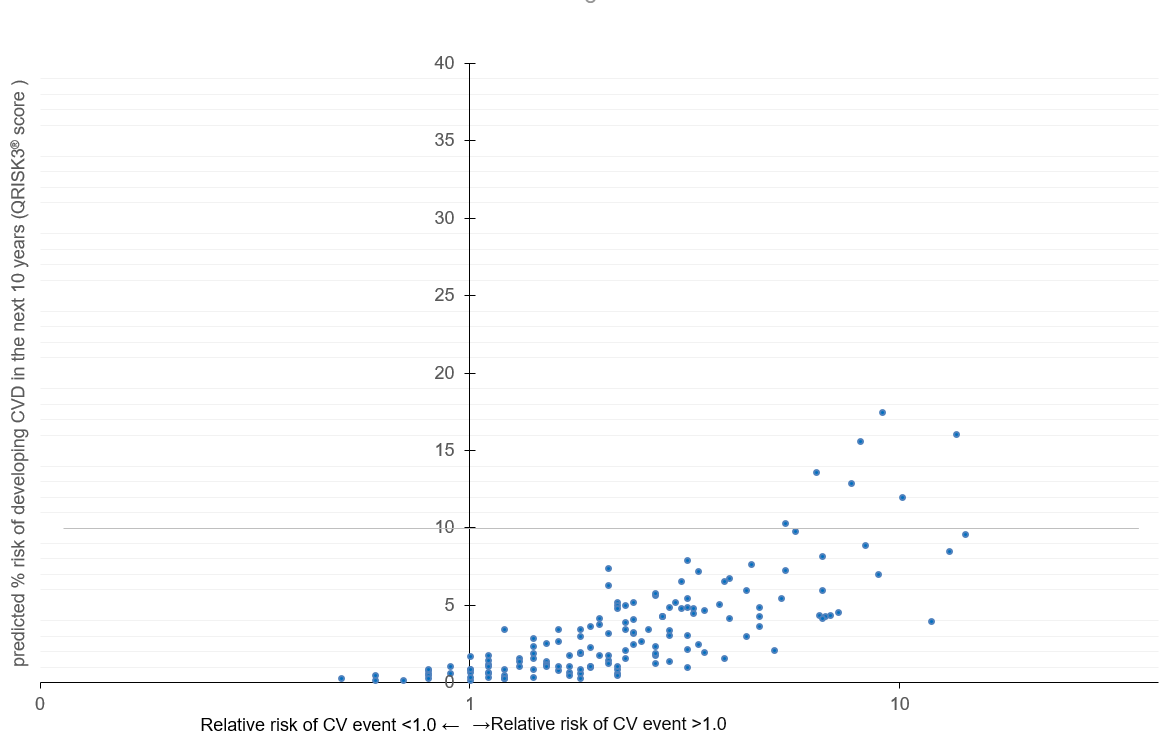


b. Premorbid predicted absolute 10 year % cardiovascular QRISK3 score vs relative risk score in all patients who had a vascular event aged 30-44 years in those who would not be eligible for NHS health check (n=56)


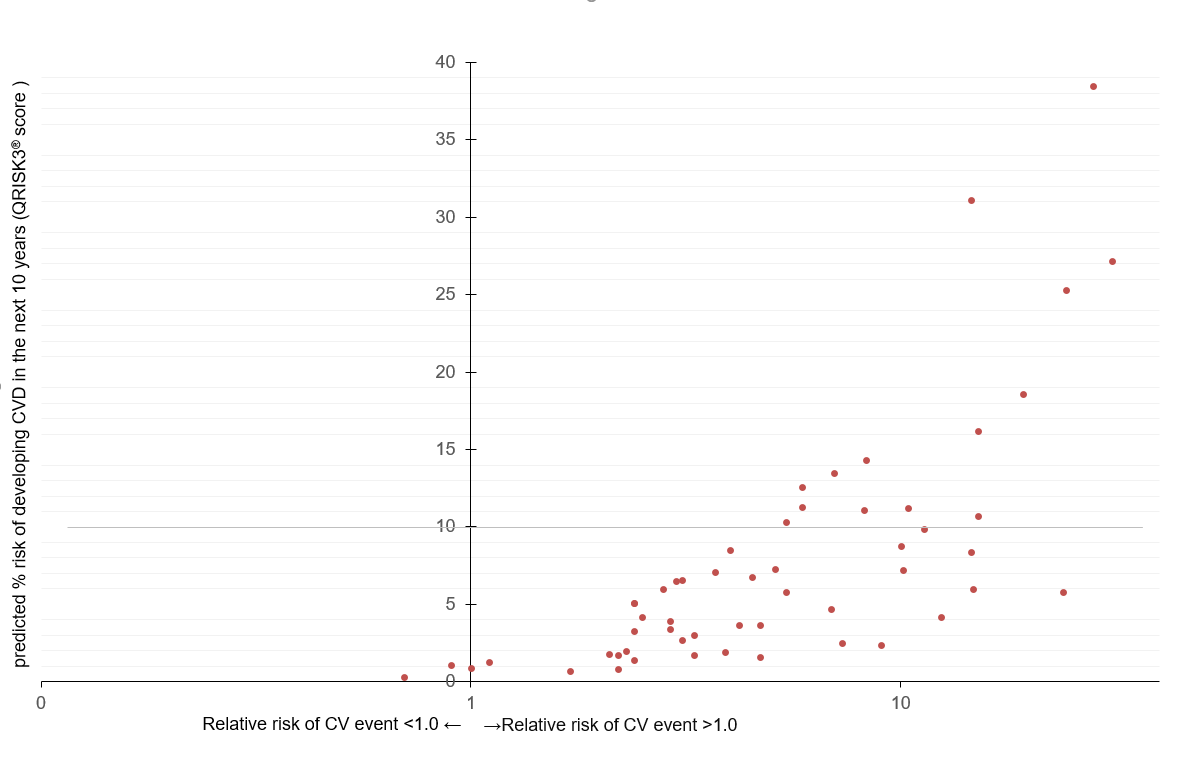


NICE 10% treatment threshold for primary prevention
