# Supplementary material for: Potential of earlier primary care health checks for prevention of cardiovascular events in younger age groups: population-based study in the United Kingdom
Source: BMC Med. 2026 Jan 24;24:107. doi: 10.1186/s12916-026-04657-7 (PMC12914909; doi:10.1186/s12916-026-04657-7)
Supplement: Supplementary file 2 — Additional file 2. Case Vignettes. [file 12916_2026_4657_MOESM2_ESM.docx]

**ADDITIONAL FILE 2- Case Vignettes**

Below are two cases, adapted for anonymity, illustrating the predicted risk scores in these female participants.

Case 1: A 39 year old Caucasian woman has a family history of coronary heart disease (her mother died of an MI aged 45). She is morbidly obese and smokes more than 20 cigarettes per day. Her systolic blood pressure is 122mHg, with standard deviation of 7 from 4 previous readings during the preceding 5 years (range 116-132mmHg), and her cholesterol ratio was 7.33. She does not have any of the other QRISK3 risk factors. Her 10 year QRISK3 score is “low” (6.5%) however her healthy heart age is 63 years and her lifetime risk of CVD is 65%.

Case 2: A 38 year old lady of Bangladeshi ethnicity has no family history of coronary heart disease in a first degree relative, she is an ex- smoker, and her blood pressure is 138mmHg, with standard deviation of 14.41 from 20 previous readings in the past 5 years. Her BMI is normal, her cholesterol ratio is 6.45, and she has none of the other QRISK3 risk factors. Her 10 year QRISK3 score is “low” (1.7%) however her healthy heart age is 45 years and her lifetime risk of CVD is 58%.

|  | CASE VIGNETTE 1 | CASE VIGNETTE 2 |
| --- | --- | --- |
| 1. Age (years) | 39 | 38 |
| 2. Sex | Female | Female |
| 3. Ethnic origin | White | Bangladeshi |
| 4. Deprivation | IMD decile 5 | IMD decile 7 |
| 5. Systolic blood pressure (mmHg) | 120mmHg | 138mmHg |
| 6. Body Mass Index (BMI) | 43.2* | 21.6 |
| 7. Total cholesterol: high density lipoprotein cholesterol ratio | 7.33 | 6.45 |
| 8. Smoking status | Heavy smoker | Ex-smoker |
| 9. Family history of coronary heart disease | mother fatal MI <50 | no |
| 10. Diabetes | no | no |
| 11. Treated hypertension | no | no |
| 12. Atrial fibrillation (atrial fibrillation, atrial flutter, paroxysmal atrial fibrillation) | no | no |
| 13. Rheumatoid arthritis | no | no |
| 14. Chronic kidney disease stage 3 or higher | no | no |
| 15. Migraine | no | no |
| 16. Corticosteroid use | no | no |
| 17. Systemic Lupus Erythematosus (SLE) | no | no |
| 18. Atypical antipsychotic use | no | no |
| 19. Severe mental illness | no | no |
| 20. Erectile dysfunction | no | no |
| 21. Blood pressure variability (SD of systolic blood pressure) | 7.0 | 14.4 |
| **10-year QRISK3 CVD Score** | **6.5%** | **1.7%** |
| **The score of a healthy person with the same age, sex, and ethnicity** | **0.7%** | **0.9%** |
| **Relative risk**** | **9.1** | **2.0** |
| **Healthy Heart Age***** | **63** | **45** |
| **Lifetime risk ******** | **65%** | **58%** |

* This is outside of the range of the QRISK3 BMI calculator, and a value of 40 kg/m^2^ was substituted

** Relative risk score: the score of a “healthy” age, sex and ethnic matched individual, i.e. with no adverse clinical indicators and a cholesterol ratio of 4.0, a stable systolic blood pressure of 125, and BMI of 25.

***Healthy Heart Age” the age at which a “healthy person” (as above) would have the same QRISK CVD Score

****QRISK Lifetime risk score; estimated risk of getting cardiovascular disease by age 99.
